# Relapsing Multiple Myeloma Presenting as Cardiac Tamponade and Obstructive Uropathy

**DOI:** 10.7759/cureus.14503

**Published:** 2021-04-15

**Authors:** Bright E Izekor, Pruthali Kulkarni, Priscilla R Powell, James Hall, Rex C Garland

**Affiliations:** 1 Internal Medicine, Baylor Scott & White Medical Center - Temple, Temple, USA; 2 Pathology, Baylor Scott & White Medical Center - Temple, Temple, USA; 3 Hematology and Medical Oncology, Baylor Scott & White Medical Center - Temple, Temple, USA

**Keywords:** myeloma, tamponade, pericardiocentesis, plasmacytoma, relapsing multiple myeloma

## Abstract

Cardiac tamponade is a rare manifestation of relapsing extramedullary multiple myeloma and portends poor prognosis. No cases of cardiac tamponade with co-occurring renal obstruction from plasmacytoma have been reported in the literature, making this case a unique presentation of relapsing multiple myeloma. The presence of known malignancy should not limit our differential diagnosis when evaluating patients with signs of cardiac tamponade.

## Introduction

Multiple myeloma is a neoplasm of clonal plasma cells. It is estimated to affect over 120,000 people in the US between 2012 and 2016 [1] and accounts for a little over 10% of all hematological malignancies [2]. While multiple myeloma is primarily a disease of the bone marrow, extramedullary manifestation has been reported in 6-20% of cases and has been associated with poorer prognosis [3]. Cardiac involvement of extramedullary multiple myeloma has been documented. Cardiac tamponade, though rare, is a very important manifestation of extramedullary multiple myeloma; only 28 cases were reported as of January 2019, with mean survival being as low as six weeks [4]. Given the rarity of myeloma-related cardiac tamponade and the dismal prognosis it portends, it is imperative that this disease be extensively described for better understanding, more effective management, and improved survival. We present here a case of cardiac tamponade in an African American male with immunoglobulin A (IgA) kappa multiple myeloma who also had an intraluminal plasmacytoma completely obstructing his left renal collecting system.

## Case presentation

A 59-year-old male from the Caribbean with a history of coccidioidomycosis, diabetes, hypertension, and thyroid goiter initially presented September 2020 with left shoulder pain. Imaging showed a pathologic fracture as well as an impending left femoral neck fracture status post-open reduction and internal fixation of the left humerus and intramedullary nail of the left femoral neck. The remainder of his baseline imaging revealed multiple lytic lesions throughout the skeleton, soft tissue masses surrounding both kidneys, a right adrenal mass, and stable miliary lung nodules with improvement of a known left upper lobe cavitary lesion. Biopsy of the left humerus and left femur showed a kappa-restricted plasma cell neoplasm. His serum protein electrophoresis (SPEP)/serum immunofixation showed a triclonal gammopathy (monoclonal protein bands at 0.4, 2.2, and 0.3 gm/dL), IgA of 3,408 mg/dL, kappa free light chains of 412 mg/L, Lactate dehydrogenase (LDH) of 1,242 IU/L, beta-2 microglobulin of 3.9 mg/L, albumin of 3.9 g/dL, and 24-hour urine immunofixation with 163 mg of protein with a kappa free light chain noted. Bone marrow biopsy showed cellularity of 60%, kappa-restricted plasma cells accounting for 6-8% with trilineage maturation, and cytogenetics being high risk with gain of 1q.

He was started on induction therapy with lenalidomide 25 mg on days 1-14 every 21 days, bortezomib 1.3 mg/m^2^ on days 1, 4, 8, and 11 of each cycle, and dexamethasone 40 mg weekly. After his first cycle, IgA improved to 1,976 mg/dL and kappa free light chains improved to 98.6 mg/L. He completed four cycles of this regimen with incorporation of zoledronic acid every 12 weeks. Following his fourth cycle, IgA levels decreased to 597 mg/dL while kappa free light chains was 145.2 mg/L. The plan was for him to complete an additional two cycles of bortezomib and dexamethasone prior to consolidative bone marrow transplant.

**Figure 1:**
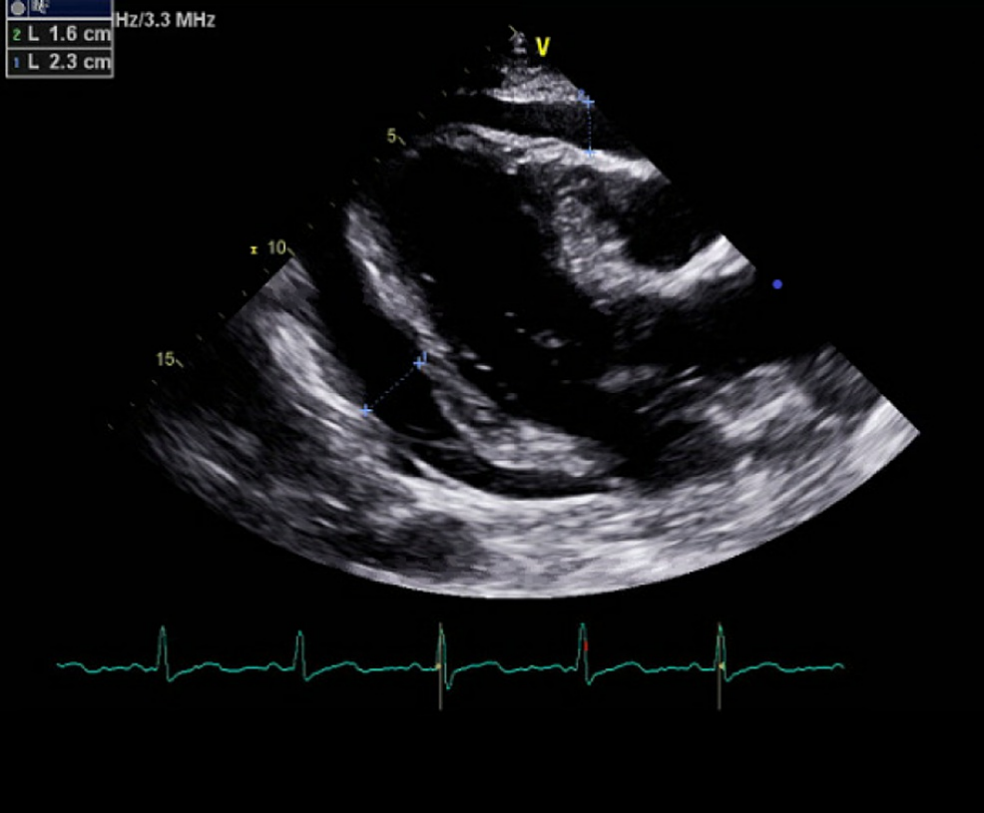
Echocardiogram showing large pericardial effusion

He presented to the hospital two weeks after his fifth cycle of chemotherapy with acute hypoxic respiratory failure. On examination, he had marked jugular venous distension and muffled heart sounds. Oxygen saturation was 85% with some improvement with supplemental oxygen, pulse was as high as 132 bpm, and blood pressure (BP) at presentation was 107/80 (baseline BP between 130s-150s systolic and 80s-90s diastolic from review of outpatient records). Workup was significant for evidence of tumor lysis syndrome (TLS) with potassium of 5.4 meq/L, LDH of 908 IU/L, phosphorus of 5.8 mg/dL, uric acid of 13.1 mg/dL, creatinine (Cr) of 1.91 mg/dL (with baseline 1.2-1.3 mg/dL), and serum calcium of 9.0 mg/dL. TLS was managed with rasburicase, allopurinol, and IV fluids. His remaining labs were significant for IgA of 1,458 mg/dL, kappa free light chains of 1,397.38 mg/L, and serum viscosity of 1.9. CT of the abdomen and pelvis without contrast showed a moderate-to-large pericardial effusion and also showed that the left kidney was diffusely increased in size with markedly increased soft tissue masses compared to previous imaging and increased density within the renal collecting system and ureter. Transthoracic echocardiogram revealed an ejection fraction of 55-62% with pericardial effusion, diastolic right ventricle (RV) collapse, right ventricular compression, and dilation of the inferior vena cava (IVC) (Figure 1).

**Figure 2:**
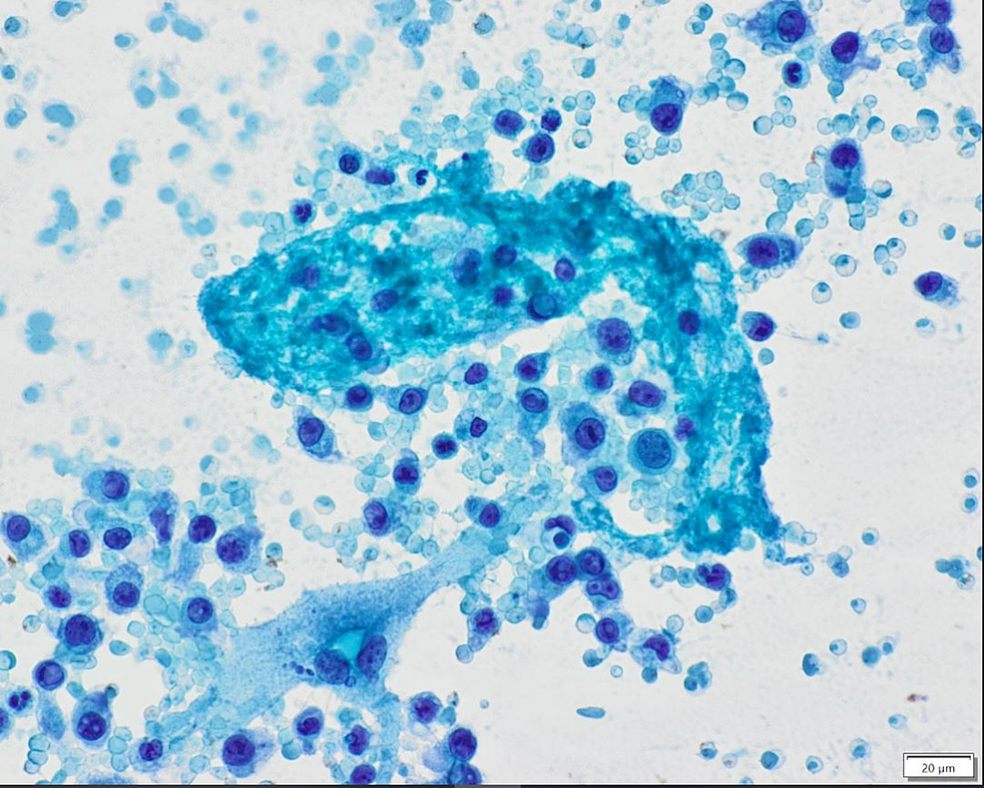
Cytology showing atypical plasmacytoid population in pericardial fluid, Papanicolaou stain, 400x

Pericardiocentesis was performed with 700 mL of dark serosanguinous fluid removed. He had significant improvement in clinical status thereafter with Cr down to 1.3 mg/dL 48 hours after the procedure, meanwhile serum Cr was 2.48 mg/dL that morning prior to pericardiocentesis. The patient’s BP also rose to 139/95 within an hour after pericardiocentesis. He was off supplemental oxygen less than 48 hours following the procedure with oxygen saturation in the mid to high 90s. Pericardial fluid LDH was above assay limit of 3,325 mg/dL and serum LDH was 1,063 mg/dL the morning before pericardiocentesis. Cytology of pericardial fluid showed cellular proliferation of discohesive plasmacytoid cells, with a subset displaying less mature features such as high nuclear-to-cytoplasmic ratio, prominent nucleoli, and more dispersed chromatin. Scattered so-called Dutcher bodies or intranuclear inclusions were also seen (Figure 2).

**Figure 3:**
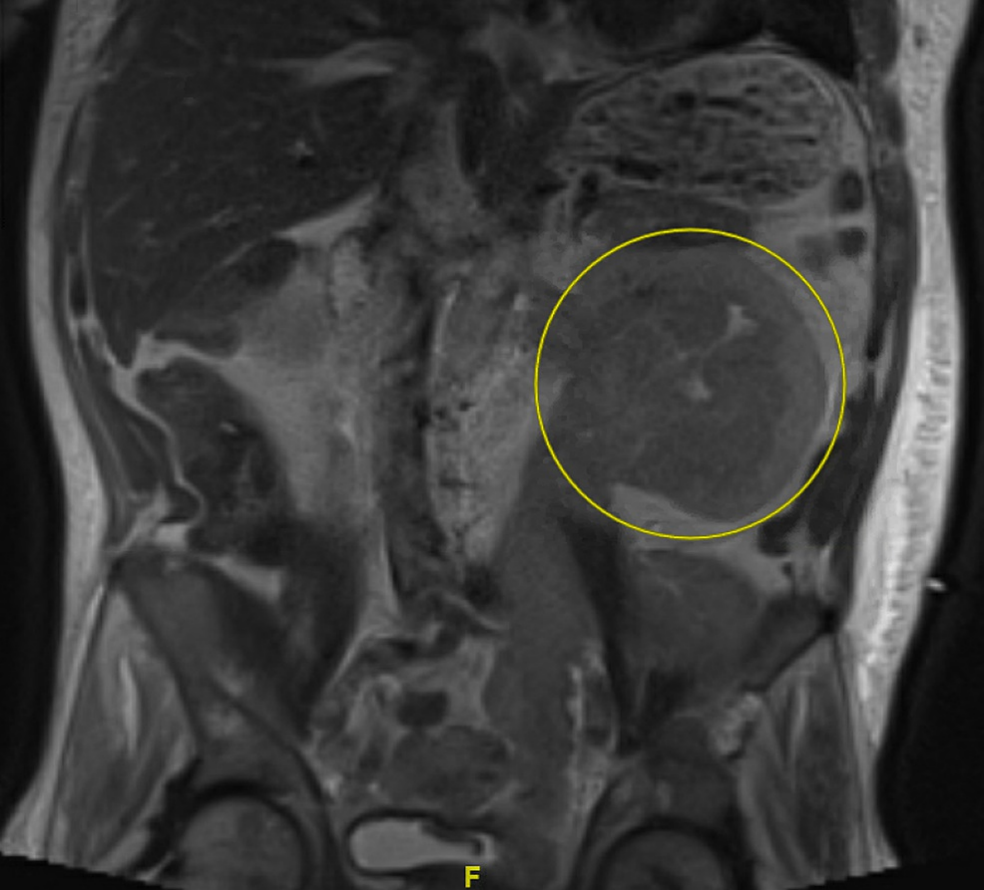
Infiltrative left renal mass highlighted with hydronephrosis

The patient underwent MRI to evaluate left kidney findings, which showed an infiltrative mass involving the entirety of the left upper collecting structures, renal pelvis, and left ureter with left-sided hydronephrosis (Figure 3).

**Figure 4:**
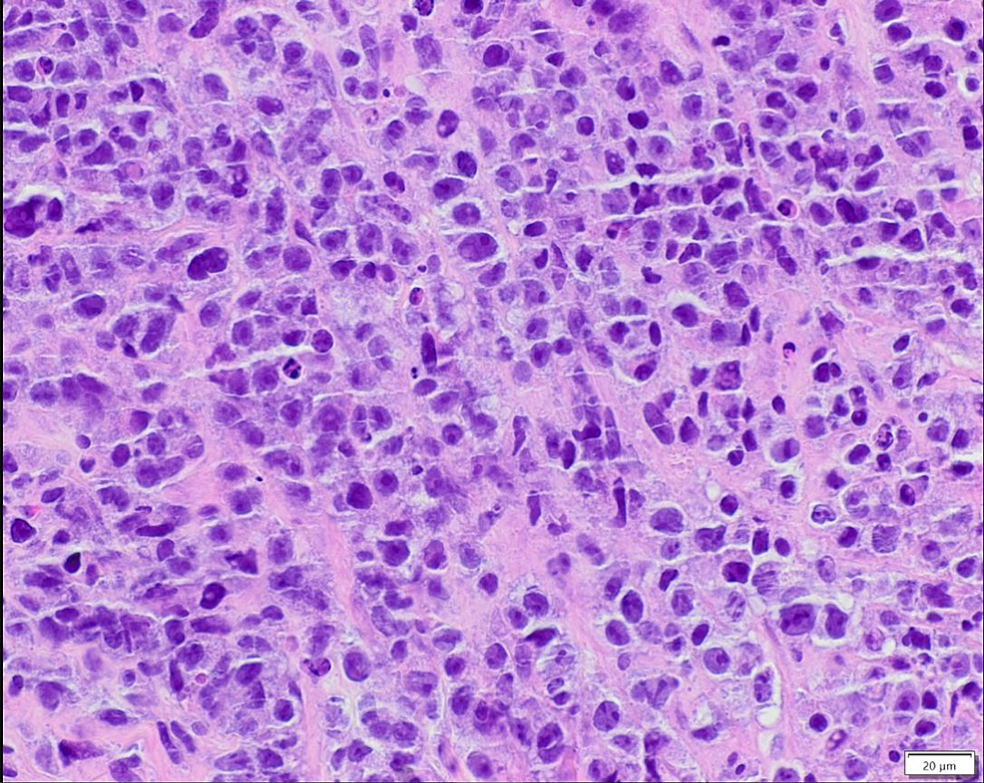
Renal biopsy showing pleomorphic cells with enlarged nuclei and prominent nucleoli, H&E, 400x

Core biopsy of the kidney demonstrated similar morphology as the pericardial fluid. There were sheets and cords of plasmacytoid cells, some of which were immature appearing with large nuclei, prominent nucleoli, and frequent apoptosis (Figure 4).

Immunostaining of the cell block from pericardial fluid and renal core biopsy were positive for CD138 and highlighted kappa-restricted plasma cells. For treatment of his aggressive light chain myeloma, he was given dexamethasone 40 mg by mouth daily, cisplatin 10 mg/m^2^/day, doxorubicin 10 mg/m^2^/day, cyclophosphamide 400 mg/m^2^/day, and etoposide 40 mg/m^2^/day continuously over four days and was subsequently able to be discharged home. Kappa free light chains were down to 68.6 mg/dL and IgA at 1,134 mg/dL at his next follow-up visit one week after discharge.

## Discussion

We presented here a case of extramedullary multiple myeloma presenting as cardiac tamponade and obstructive plasmacytoma. Extramedullary multiple myeloma occurs when malignant plasma cells detach from the bone marrow and deposit in other tissues. While the exact mechanisms of detachment remain largely unknown, some mechanisms proposed include decreased expression of adhesion molecules such as VLA-4 and CD-44, loss of CD56, decreased expression of P-selecting, and downregulation of tetraspanins [5-7]. Following detachment, these cells then spread, either hematologically or through direct extension from skeletal tumors, and are deposited in tissues where they form plasmacytomas [8].

Cardiac tamponade from extramedullary disease is rare, with only 28 cases reported as of January 2019 [4]. This patient met most of Beck’s triad with jugular venous distension and muffled heart sounds on physical examination [9]; BP was lower than baseline, although the patient was not overtly hypotensive. This patient also presented with hypoxic respiratory failure not typical of cardiac tamponade; it was theorized that the patient’s hypoxia was likely multifactorial with cardiac tamponade (poor RV output) with his underlying lung disease playing a significant role. The fact that his hypoxia resolved following pericardiocentesis supports our theory that it is likely related to cardiac tamponade. Other possible causes are pneumonia, pulmonary embolism, and pulmonary edema from vascular endothelial dysfunction due to acute illness. Echocardiographic evidence of tamponade included significant mitral and tricuspid inflow respiratory variation, RV compression, RV collapse, and dilated IVC in the setting of large circumferential pericardial effusion.

Pericardial fluid analysis was consistent with malignant pericardial effusion. The mechanism of pericardial involvement in extramedullary multiple myeloma is yet to be described in the literature. It was theorized in this case that it was likely secondary to hematological spread. It was considered that the malignant cells found on cytology could have been cells leaked from peripheral blood; this was however considered unlikely as 1) no large vessels were punctured during the procedure, 2) pericardial fluid was not consistent with hemopericardium, and 3) pericardial fluid LDH was over three times that of serum. This supports the theory that those malignant cells were likely seeded prior and had proliferated in the pericardial space, precipitating a malignant pericardial effusion. Other malignancies associated with pericardial effusion and tamponade are cancers of the lung, breast, ovary, prostate, colon, gastric, kidney, and bladder, lymphoma, leukemia, and malignant melanoma [10,11]. Regardless of the etiology, diagnosis of cardiac tamponade warrants urgent management to prevent hemodynamic collapse. The European Society of Cardiology (ESC) and the American Heart Association (AHA) recommend pericardiocentesis as the preferred management for cardiac tamponade [12-14]. This patient did undergo pericardiocentesis under echocardiography with prompt resolution of his symptoms.

This patient also presented with plasmacytoma completely obstructing his left kidney. Initially, this was thought to be the principal contributor to his acute kidney injury (AKI). However, given that only his left kidney was affected and that his AKI improved to baseline following pericardiocentesis, it was then determined that his AKI was more likely due to hypoperfusion from cardiac tamponade and/or TLS. Other possible causes of AKI in patients with multiple myeloma are hypercalcemia, cast nephropathy, monoclonal Ig deposition disease, light chain amyloidosis, and glomerulonephritis [15].

The co-occurrence of obstructing renal plasmacytoma with malignant pericardial effusion is a unique presentation of relapsing multiple myeloma. From our literature review, no prior cases have been reported. A single case of Castleman’s disease with renal infiltration and cardiac tamponade was reported by Kim et al. in 2012 [16]. While cardiac tamponade is known to portend a dismal prognosis, the impact of concomitant tamponade and renal plasmacytoma on prognosis remains unclear; one can only assume that the prognosis is likely worse than tamponade alone.

## Conclusions

In conclusion, cardiac tamponade could be the principal presenting sign of relapsing multiple myeloma; it portends poor prognosis. Patients with a history of multiple myeloma presenting with dyspnea, jugular venous distension, and muffled heart sounds should be evaluated for cardiac tamponade. This is true especially if they have signs of hemodynamic compromise; the presence of known malignancy should not limit our differential diagnosis.
